# Mitochondrial dysfunctions induce PANoptosis and ferroptosis in cerebral ischemia/reperfusion injury: from pathology to therapeutic potential

**DOI:** 10.3389/fncel.2023.1191629

**Published:** 2023-05-24

**Authors:** Ruining She, Danhong Liu, Jun Liao, Guozuo Wang, Jinwen Ge, Zhigang Mei

**Affiliations:** ^1^Key Laboratory of Hunan Province for Integrated Traditional Chinese and Western Medicine on Prevention and Treatment of Cardio-Cerebral Diseases, College of Integrated Traditional Chinese and Western Medicine, Hunan University of Chinese Medicine, Changsha, Hunan, China; ^2^Medical School, Hunan University of Chinese Medicine, Changsha, Hunan, China; ^3^Hunan Academy of Traditional Chinese Medicine, Changsha, Hunan, China; ^4^Third-Grade Pharmacological Laboratory on Chinese Medicine Approved by State Administration of Traditional Chinese Medicine, China Three Gorges University, Yichang, Hubei, China

**Keywords:** ischemic stroke, cerebral ischemia/reperfusion injury (CI/RI), mitochondrial dysfunctions, PANoptosis, PANoptosome, ferroptosis

## Abstract

Ischemic stroke (IS) accounts for more than 80% of the total stroke, which represents the leading cause of mortality and disability worldwide. Cerebral ischemia/reperfusion injury (CI/RI) is a cascade of pathophysiological events following the restoration of blood flow and reoxygenation, which not only directly damages brain tissue, but also enhances a series of pathological signaling cascades, contributing to inflammation, further aggravate the damage of brain tissue. Paradoxically, there are still no effective methods to prevent CI/RI, since the detailed underlying mechanisms remain vague. Mitochondrial dysfunctions, which are characterized by mitochondrial oxidative stress, Ca^2+^ overload, iron dyshomeostasis, mitochondrial DNA (mtDNA) defects and mitochondrial quality control (MQC) disruption, are closely relevant to the pathological process of CI/RI. There is increasing evidence that mitochondrial dysfunctions play vital roles in the regulation of programmed cell deaths (PCDs) such as ferroptosis and PANoptosis, a newly proposed conception of cell deaths characterized by a unique form of innate immune inflammatory cell death that regulated by multifaceted PANoptosome complexes. In the present review, we highlight the mechanisms underlying mitochondrial dysfunctions and how this key event contributes to inflammatory response as well as cell death modes during CI/RI. Neuroprotective agents targeting mitochondrial dysfunctions may serve as a promising treatment strategy to alleviate serious secondary brain injuries. A comprehensive insight into mitochondrial dysfunctions-mediated PCDs can help provide more effective strategies to guide therapies of CI/RI in IS.

## Graphical Abstract

Overview of mitochondrial dysfunctions-induced PANoptosis and ferroptosis during CI/RI. 
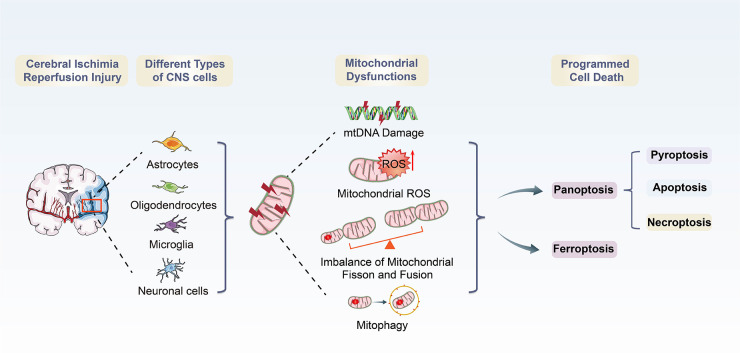

## Introduction

Stroke is the second leading cause of death and the third major cause of disability globally (Pandian and Sebastian, 2021). In 2019, around 12.2 million people suffered from a new or recurrent stroke, which has increased substantially from 1990 to 2019 (Zhou et al., 2016; GBD 2019 Stroke Collaborators, 2021). Ischemic stroke (IS) accounts for more than 80% of all stroke types, and according to incomplete statistics, about 14 million people suffer from IS annually (Farina et al., 2021). During IS, blood flow is blocked, oxygen and nutrients are depleted, triggering a cascade of ischemic events in the brain (Magistretti and Allaman, 2015; Shen et al., 2021; Shi et al., 2021). Treatment for IS can be achieved through reperfusion, which restores blood flow/oxygenation to the brain in a timely fashion and efficiently salvaging the function of potentially reversible ischemic penumbra by thrombolysis such as intravenous recombinant tissue-type plasminogen activator (rtPA) or mechanical thrombectomy. Rapid reperfusion paradoxically has the constraint of a short recanalization time window and may result in irreparable neurological damage, a condition known as cerebral ischemia/reperfusion injury (CI/RI) (Gao et al., 2015).

Although recent clinical trials have shown that the administration of reperfusion therapy 24 h or more after stroke onset has a positive effect on the prognosis of patients with acute ischemic stroke (Ragoschke-Schumm and Walter, 2018), it often leads to additional cerebral damage, creating an important clinical dilemma. Reperfusion-induced reactive oxygen species (ROS) production overwhelms the cell’s anti-oxidative defense mechanism, rendering it incapable of scavenging free radicals, disturbing neuronal homeostasis, which leads to inflammatory response, oxidative stress, apoptosis, necrosis, and other pathological processes, culminating in cell death (Deb et al., 2010).

Increasing evidence indicates that mitochondria play vital roles in improving neuronal survival and neurological function after IS (Deb et al., 2010; Anzell et al., 2018; Yang et al., 2021). Mitochondria are cellular organelles responsible for energy production and metabolism in cells, providing adenosine triphosphate (ATP) to active neurons (Sjostrand, 1953; Gustafsson et al., 2016; Xian and Liou, 2021). During CI/RI, energy balance is disturbed due to reduced blood supply and ATP synthesis is disturbed (Borutaite, 2010). One of the hallmarks of CI/RI are mitochondrial dysfunctions (Vosler et al., 2009), which are characterized by mitochondrial oxidative stress, mitochondrial Ca^2+^ overload, iron dyshomeostasis, mitochondrial DNA (mtDNA) defects and mitochondrial quality control (MQC) disruption. Following ischemia and reperfusion, mitochondrial dysfunctions initiate a cascade of events that result in acute and persistent inflammatory responses and activate the programmed cell deaths (PCDs), such as ferroptosis and PANoptosis, a recently proposed concept of programmed cell deaths characterized by a unique inflammatory cell death modality, including pyroptosis, apoptosis and necroptosis. These pathophysiological processes are intertwisted and deleterious to the neural cells, regulating the disease and immune response of CI/RI. However, how mitochondrial dysfunctions govern cell death has been unclear and somewhat controversial. A thorough understanding of mitochondrial dysfunctions in different physiological and pathological conditions is essential to provide therapeutic avenues for IS (Bock and Tait, 2020).

This review will offer an insight into the pathomechanism underlying the mitochondrial dysfunctions in various types of PCDs and mitochondria-targeted therapeutic potential against PCDs especially PANoptosis and ferroptosis in CI/RI. Clarifying the relationship of pathology between mitochondrial dysfunctions and PCDs and uncover the molecular pathways will not only contribute to a thorough understanding of the mitochondrial dysfunction-mediated PCDs machinery but also lighten potential novel pharmacological targets for IS.

## 2. CI/RI and mitochondrial dysfunctions

### 2.1. CI/RI pathology

The earliest symptom of IS is cerebral ischemia (Khoshnam et al., 2017). Protecting the ischemic penumbra and restoring brain function is critical. However, CI/RI is inevitable after restoration of blood flow and may be the most important determinant of poor prognosis (Kalogeris et al., 2016; Nentwich, 2016). Reperfusion produces paradoxical tissue responses, which lead to a severe imbalance of metabolic supply and demand, and eventually activates neuronal death and causes hippocampal and cortical damage, initiating cerebral hemorrhage and deteriorating the blood-brain barrier (BBB) (Kishimoto et al., 2019; Huang et al., 2021). CI/RI results from a complex series of pathophysiological events including burst of ROS, free radical damage, Ca^2+^ homeostasis disorder, EAA toxicity, neuroinflammation, and fat decomposition, etc., (Kalogeris et al., 2016; Wu M. et al., 2021). Ca^2+^ overload and ROS burst are the initial events of CI/RI (Pundik et al., 2012; Hayyan et al., 2016; Kalogeris et al., 2016; Wu M. Y. et al., 2018; Chen and Li, 2020).

During cerebral ischemia, ATP synthesis efficiency declines due to ischemia and hypoxia in the brain, along with acidic intracellular metabolites and purine bases surging. Thus, the Na^+^/H^+^ exchanger (NHE) exchanges for sodium ions (Sansbury and Spite, 2016) and the lack of oxygen supply forces cells to produce ATP, which is insufficient to maintain ATPases (e.g., Na^+^/K^+^ ATPase) function. This results in cellular Ca^2+^ overload and disruption of mitochondrial architecture. Once reperfusion, the oxygen and substrates required for aerobic ATP generation are restored, and hydrogen ions that accumulate in the extracellular space are removed, which promotes additional Ca^2+^ influx. At the same time, the oxygen influx could also fuel ROS production (Chen H. et al., 2011). Ca^2+^ accumulation also mediates the excitotoxicity and then promotes cerebral edema and activation of the intracellular self-destruction cascade. Mitochondria absorb excess Ca^2+^ when Ca^2+^ levels are elevated by excitotoxicity, leading to organelle enlargement and formation of the mitochondrial permeability transition pore (mPTP), which executes and activates cell death pathways (Andrabi et al., 2020). Reperfusion-promoted ROS damage and oxidative stress injure the proteins, lipids, as well as mtDNA, which causes straight damage to mitochondrial function after CI/RI (Borutaite, 2010). Moreover, CI/RI seriously affects glial cells, including oligodendrocytes, microglia, and astrocytes (Song et al., 2017). Oligodendrocytes are particularly sensitive to injuries, including hypoxia, ROS/nitrogen species (NOS) and excitotoxicity (Merrill and Scolding, 1999; Smith et al., 1999), which impair the functional activity of the mitochondrial respiratory chain (Ziabreva et al., 2010). Microglia are critical for regulating neuroinflammation. Mitochondrial dyshomeostasis injures microglial function and exacerbated the pathogenic process of IS (Zhou et al., 2019). Astrocytes communicate and protect neurons from hypoxia and excitotoxicity through the gap junction and BBB, whereas inhibition of astrocyte mitochondrial function leaves neurons vulnerable to cell death (Ouyang et al., 2013). Mitochondrial dysfunctions are the most critical link of CI/RI.

### 2.2. Mitochondrial abnormalities and dysfunctions in CI/RI

**FIGURE 1:**
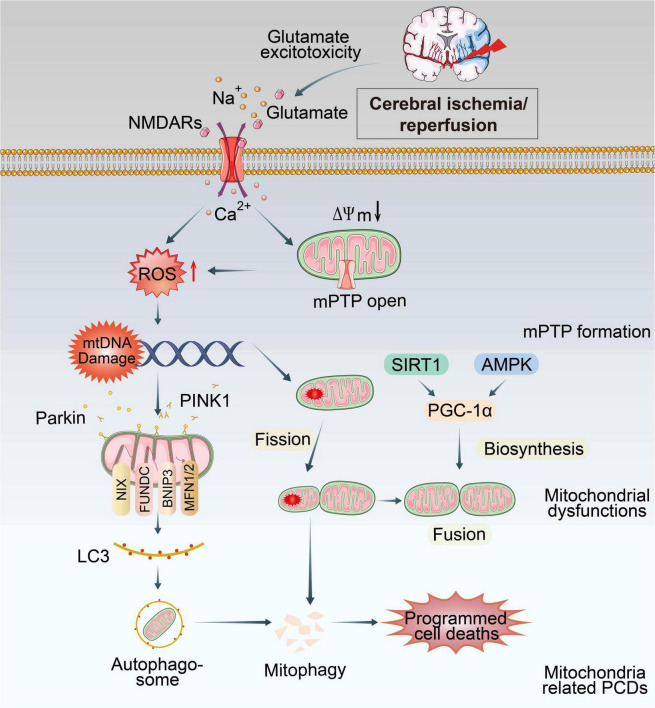
Overview of mitochondrial dysfunctions during CI/RI. Mitochondria are the most susceptible organelle to CI/RI. ATP consumption, glucose/O_2_ deprivation, burst of ROS, Ca^2+^ overload, excitotoxicity, inflammatory response and other consequences of CI/RI could trigger mitochondrial dysfunctions including mitochondrial oxidative stress, Ca^2+^ overload, iron dyshomeostasis, mitochondrial DNA defects, mitochondrial quality control disruption as well as mitochondrial-induced PCDs. These cellular processes ultimately lead to the death of neuron.

Mitochondria are the most vulnerable organelle to cerebral ischemic injury (Hill et al., 2018). Burst of ROS, Ca^2+^ overload, excitotoxicity and other consequences of CI/RI could trigger mitochondrial dysmorphology/dysfunctions (Figure 1). Notably, preserving or promoting mitochondrial function is a potential therapeutic target for treating CI/RI.

#### 2.2.1. Mitochondrial structure abnormalities

In the brain, mitochondria generate ATP by electron transportation chain (ETC), which is composed of transmembrane protein complexes (I-IV) embedded in the inner mitochondrial membrane (IMM) (Tang et al., 2016; Zhao et al., 2019). During cerebral ischemia, the energy supply is drastically reduced. IMM and mitochondrial cristae structure deform due to oxygen radicals and Ca^2+^ overload, triggering mitochondrial response, including excessive ROS production, mitochondrial Ca^2+^ overloading, and disrupted MQC. During reperfusion, Ca^2+^ influx and ROS burst promote the mitochondria’s swelling, increasing cytoplasmic density, depolarization of mitochondrial membrane potential (ΔΨm) and opening of the mPTP (Solenski et al., 2002).

The mPTP is a high-conductance channel that composed of three proteins: the voltage-dependent anion channel (VDAC) in the outer mitochondrial membrane (OMM), the adenine nucleotide translocator (ANT) in the IMM and cyclophilin D (CypD) in the mitochondrial matrix (Rao et al., 2014; Pérez and Quintanilla, 2017; Rottenberg and Hoek, 2017), regulating molecular exchange between the mitochondrial matrix and cytoplasm. The mPTP regulation by CypD is the most critical for mitochondrial morphology. Under normal conditions, mPTP is closed, and the IMM selectively allows the passage of small metabolic substrates and ions. When the cell undergoes oxidative stress, Ca^2+^ and ROS concentrations burst and the permeability of mPTP increases, initiating further production and release of ROS that damage both mitochondrial and nuclear DNA, proteins, and phospholipids. Further, the opening of the mPTP forms the mitochondrial permeability transition (MPT). It releases cytochrome C (Cyt C) and serine protease into the cytosol (Tajeddine, 2016), which could trigger the caspase cascade, leading to PCDs and a series of damage (Van Opdenbosch and Lamkanfi, 2019).

#### 2.2.2. Mitochondrial oxidative stress

Oxidative phosphorylation (OXPHOS) is an oxygen-dependent process in mitochondria that consumes chemical energy from catabolism to produce ATP and power energy-dependent biological processes. The OXPHOS system works through a series of protein complexes, consisting of ETC complexes I, II, III, and IV, and ATP synthase (complex V), along with two electron carriers, Cyt C, and coenzyme Q (CoQ) (Zhao et al., 2022). Thus, mitochondria are famous as “the powerhouse of the cell”.

Mitochondrial oxidative stress is a condition that arises from an imbalance between oxidation and antioxidation in the mitochondrial respiratory chain (Sinha et al., 2013). It plays an essential role in CI/RI development (Quesnelle et al., 2015). The reduction of oxygen in mitochondria following cerebral ischemia limits mitochondrial OXPHOS, which decreases ATP production, leading to the release of oxygen radicals from ETC and the eruption of incomplete metabolism such as superoxide anion (O_2_^–^), hydroxyl radicals (⋅OH), reactive nitrogen species (RNS) and nitric oxide (NO) (Vinogradov and Grivennikova, 2016; Trujillo-Rangel et al., 2022). While during reperfusion, after the oxygen supply is restored, the pro-oxidant enzyme system and mitochondria use oxygen as a substrate to produce oxygen radicals, generating transient but an exorbitant burst of ROS in cells and ultimately triggering a series of processes ranging from altered cell signaling pathways to cell death (Andrabi et al., 2020). Furthermore, the reperfusion process also significantly reduces the activity of succinic dehydrogenase and Cyt C oxidase and another key enzyme along the ETC, leading to a reduction in OXPHOS efficiency, which affects ATP production (Cadenas and Davies, 2000).

#### 2.2.3. Mitochondrial Ca^2+^ overload

Normally, cytosolic Ca^2+^ is strictly regulated through the cell membrane, endoplasmic reticulum, and mitochondria. As Ca^2+^ buffer, mitochondria absorb substantial amounts of cytosolic Ca^2+^ at the expense of ΔΨm. The pathways of Ca^2+^ entry into the mitochondrial matrix are known as the mitochondrial calcium uniporter (MCU), the “rapid mode” mechanism, and the mitochondrial ryanodine receptor (Feissner et al., 2009). When cerebral blood flow gets interrupted and oxygen supply is reduced, Na^+^/K^+^ ATPase and other ion channels are prevented from maintaining a regular electrochemical gradient, resulting in continued depolarization of glial and neuronal cells (Liao et al., 2019). Open voltage gated Ca^2+^ channels, insufficient Ca^2+^ pump activity, and ATP deficiency lead to increased intracellular Ca^2+^ concentration. Ca^2+^ overload causes the release of excitatory neurotransmitters, particularly glutamate extracellularly (Singh et al., 2019), which binds to NMDA (N-methyl-D-aspartic acid) and other ion receptors, causing massive Ca^2+^ influx and consequent excitotoxicity. Excessive matrix Ca^2+^ concentrations, especially when associated with oxidative stress, precipitate the opening of mPTP (Briston et al., 2017), which is associated with apoptosis via the mitochondrial pathway or other PCDs due to mitochondrial damage (Bernardi et al., 2022). There has also been evidence that mitochondrial Ca^2+^ uptake can be responsible for the production of free radicals (Feissner et al., 2009). The mechanism of mitochondrial Ca^2+^ overload is a topic of great debate in the field.

#### 2.2.4. MtDNA defects

Mitochondria are the only organelle possessing their circular genome, which is 16.6 kb in mammals, encoding 13 subunits that are essential in the maintenance and regulation of mitochondrial functions, such as encoding essential proteins of ETC and OXPHOS system (Liu H. et al., 2022). As mtDNA exists within the mitochondrial matrix or attached to the IMM, it can be easily damaged by free radicals produced by the respiratory chain. However, it is barely protected by histones and cannot effectively synthesize glutathione to remove oxygen radicals (Fariss et al., 2005). Following CI/RI, mitochondria activate several mtDNA repair and clearance pathways such as direct reversal (DR), DNA mismatch repair (MMR), base excision repair (BER), double-strand breaks (DSBs) and other mtDNA repair pathways (Alexeyev et al., 2013). In mitochondria, BER is the most typical mechanism to repair various types of DNA damage affecting the nuclear genome (Fontana and Gahlon, 2020). If these repair mechanisms are not sufficient to restore mtDNA structure and function, irreversible defects occur, leading to mutations in mtDNA (Liu H. et al., 2022). Furthermore, mtDNA mutations alter tRNA structure, which defects the assembly of the respiratory chain complex and enzyme activity, further increasing ROS production and exacerbating mtDNA mutations, creating a vicious cycle in CI/RI. Chen H. et al. (2001) showed that CI/RI could cause mtDNA damage. Although mtDNA can repair itself after <30 min of transient cerebral ischemia, the damage is irreversible after prolonged ischemia, which reduces the activity of complexes I and IV in the ETC, thereby disrupting the integrity of the respiratory chain complex electron transport. The mtDNA mutation also affects mitochondrial autophagy. Compared to normal cells, mtDNA mutant cells show reduced expression of autophagy marker protein light chain 3 (LC3) and reduced accumulation of autophagic substrate p62, resulting in impaired mitochondrial autophagy and significantly increased ROS levels (Zhang et al., 2018). Mitochondrial DNA damage could induce ATP synthesis defect, aggravating the outcome of programmed cell deaths, with the poor clinical symptoms such as cognitive impairment, Alzheimer’s disease (AD) and Parkinson’s disease (PD) (Anzell et al., 2018). Consequently, mtDNA is an important driver of CI/RI and can be used as a marker from primary plasma samples or tissue (Chong et al., 2022; Salvador et al., 2023). Mitochondrial diseases stemming from mtDNA point mutations and deletions present a wide clinical spectrum of phenotypes (Nissanka and Moraes, 2018). ELISA, PCR amplifications, whole-genome sequencing could be used for detection (Bernal-Tirapo et al., 2023). Further investigation of mtDNA as a potentially sensitive marker of CI/RI and response to mitoprotective therapy is warranted.

#### 2.2.5. Disrupted MQC

Mitochondrial quality control (MQC) is a significant process for maintaining mitochondrial health and function (Youle, 2019), which involves mitochondrial biogenesis, mitochondrial fission and fusion, and mitophagy (Pickles et al., 2018). These processes are essential for the production of energy, the maintenance of mitochondrial structure and function, and the removal of damaged or dysfunctional mitochondria.

##### 2.2.5.1. Mitochondrial biosynthesis

Mitochondrial biogenesis refers to the generation of new mitochondrial mass and the replication of mitochondrial DNA by proliferation of pre-existing organelles, which is essential to meet increased cellular energy demands and to repopulate mitochondrial contents in newly generated cells during cell proliferation (Yang et al., 2019).

The key regulators of mitochondrial biosynthesis include peroxisome proliferator-activated receptor-γ coactivator 1α (PGC-1α), AMP-activated protein kinase (AMPK), nuclear respiratory factor 1/2 (NRF1/2), mitochondrial transcription factor A (TFAM) and sirtuin 1 (SIRT1) (Li et al., 2021). SIRT1-PGC-1α and AMPK-PGC-1α axes are key pathways that regulate mitochondrial biogenesis (Li et al., 2017). During CI/RI, PGC-1α is activated by upstream AMPK or SIRT1 and deacetylases through phosphorylation and deacetylation modifications. Meanwhile, upregulating the mammalian target of the rapamycin (mTORC1)/PGC-1 signaling pathway could activate mitochondrial biogenesis and cellular senescence (Summer et al., 2019). PGC-1α could improve ATP production and mitochondrial mass by activating NRF1/TFAM axis in oxidative stress environments (You et al., 2016). After IS, microglial PGC-1α expression upregulates for a short period of time, significantly reducing neurological deficits after ischemic injury, with reduced neuroinflammation and enhanced mitophagy (Han et al., 2021). Meanwhile, PGC1-α is a master regulator to activate superoxide dismutase 2 (SOD2) and the uncoupling protein 2 (UCP2); both are mitochondrial proteins and may contribute to neuronal survival and ROS scavenging (Chen S. D. et al., 2011). Furthermore, PGC-1α could regulate dynamin-related protein 1 (Drp1) protein expression and phosphorylation (Peng et al., 2017). In conclusion, activation of mitochondrial biosynthesis maintains mitochondrial homeostasis. It increases cellular antioxidant and anti-infective activity and has been proposed as a potential new target for mitigating mitochondrial damage during CI/RI disease.

##### 2.2.5.2. Mitochondrial fission and fusion

Mitochondria are morphologically dynamic organelles that often undergo fission and fusion events that regulate mitochondrial integrity and bioenergetics and contribute to maintaining cellular homeostasis in healthy and diseased cells. Under normal conditions, mitochondria change shape, size and number by constantly fusing and dividing to meet the needs of cellular metabolism. However, under the induction of ischemia and hypoxia injury factors, ROS-induced mitochondrial oxidative stress can directly lead to a disruption of the relative mitochondrial fission/fusion balance, resulting in increased mitochondrial breakage and fragmentation and increased susceptibility of neurons to cell death.

In mitochondrial fission, Drp1 and fission protein 1 (Fis1) can divide mitochondria by binding to receptors on the OMM through multiple post-translational modifications, including S-nitrosylation, phosphorylation, SUMOylation, dephosphorylation, and ubiquitination (Qin et al., 2021). In CI/RI conditions, an increase in ROS levels disrupts mitochondrial membrane potential and mitochondrial depolarization, resulting in the translocation of Drp1 to the OMM via the recruitment of mitochondrial Fis1, fission factor (MFF) and mitochondrial dynamics proteins of 49/51 kDa (MiD49/MiD51), also known as MIEF1/MIEF2, where it promotes excessive mitochondrial fragmentation by coupling guanosine triphosphate (GTP) hydrolysis (Estaquier and Arnoult, 2007). Drp1-mediated mitochondrial fission is an initial event required for ischemic neuronal cell death (Fonseca et al., 2019). It has been suggested that the proapoptotic B-cell leukemia/lymphoma 2 (Bcl-2) family protein Bax function directly or indirectly as a Drp1 receptor to promote mitochondrial fission and cell death (Montessuit et al., 2010). Liu et al. (2012) found that Drp1 and P-Drp1 upregulation occurred after tMCAO, peaking at 2 and 14 days, respectively, suggesting an increase in mitochondrial fission in I/R condition. *In vitro* and *in vivo* studies shows that the mitochondrial fission inhibition by the Drp1 inhibitor or siRNA had beneficial effects on cerebral ischemia (Grohm et al., 2012; Flippo et al., 2020). Drp1 inhibition may have therapeutic value in treating stroke and neurodegeneration.

Mitochondrial fusion shares the mitochondrial matrix or metabolites such as proteins, mtDNA, or membrane components where the ETC occurs (Anzell et al., 2018). At the same time, damaged mitochondria can be repaired through fusion with healthy mitochondria to integrate contents and promote cell survival by complementation (Liu et al., 2018). Fusion proteins, including optic atrophy 1 (OPA1) and mitofusin 1/2 (MFN1/2), can protect tissues and neurons from death under CI/RI through their pro-fusion function (Dimmer and Scorrano, 2006). MFN1 and MFN2 proteins, which contain two transmembrane domains in the OMM with a GTPase domain, provide energy for OMM fusion by mixing the mitochondrial lipid bilayer. Similarly, OPA1 performs a similar function to enable IMM fusion (El-Hattab et al., 2018). The short form of OPA-1 (S-OPA1) mediates inner mitochondrial membrane fission, while the long form of OPA-1 (L-OPA1) has been reported to protect ischemic injuries by maintaining mitochondrial functions and attenuating neuronal apoptosis (Cipolat et al., 2004). Moreover, elevated levels of MIEFs promote in a manner that is mediated by MFN1/2 and OPA1 but independent of Drp1, and MIEF1/2 can alleviate hFis1-induced mitochondrial fragmentation and contribute to mitochondrial fusion (Yu et al., 2021). In hypoxic situations, CI/RI can impair mitochondrial fusion by decreasing OPA1 or depleting MFN2, thereby undermining intracellular homeostasis and inducing neuronal death (Peng et al., 2018; Wei et al., 2019; Chen Y. et al., 2020). Under I/R conditions, the upregulation of OPA1 expression greatly facilitates mitochondrial fusion, reversing the interconnected mitochondrial morphology and alleviates I/R-induced neuronal apoptosis, thereby reducing infarct volume (Wei et al., 2019). Furthermore, the downregulation of MFN2 aggravated the CI/RI by inhibiting autophagosome formation and the fusion of autophagosomes and lysosomes, demonstrating that MFN2 could ameliorate CI/RI by promoting autophagy (Peng et al., 2018).

Therefore, a delicate dynamic balance between fission and fusion is essential to maintain the structure and function of mitochondria (Li and Liu, 2018; Zhang et al., 2019). Excessive mitochondrial fission or insufficient fusion promotes the decreased ATP production and mtDNA stability, impaired mitochondrial permeability transition pore sensitivity, and cell death (Chen et al., 2010; Zhou et al., 2017; Wei et al., 2019).

##### 2.2.5.3. Mitophagy

Mitochondrial autophagy, also known as mitophagy, is a cellular process that selectively removes the dysfunctional and damaged mitochondria by coordinated mitophagy pathways. Under ROS stress, cell aging, nutritional deprivation, and other conditions, mitochondrial depolarization damage will manifest. To preserve the integrity of the mitochondrial network and restore cellular homeostasis, an autophagy system is activated to encase and degrade dysfunctional mitochondria selectively. This mechanism consists primarily of four steps (Xu et al., 2020): 1) External stimuli dissipate mitochondria and disrupt mitochondrial membrane potential (MMP), which is the prerequisite for mitophagy to occur. 2) Mitochondrial autophagosomes take shape. 3) Mitochondrial autophagosomes are delivered to the lysosome for degradation. 4) Lysosomes degrade mitochondrial contents. In mammalian cells, PINK1/Parkin axis is one of the most studied mitophagy mechanisms. The serine/threonine kinase PINK1 and the E3 ubiquitin ligase Parkin cooperatively sense cellular stress and promote the binding of ubiquitinated proteins to microtubule-associated protein LC3 to form autophagosomes and then initiate the autophagy mechanism (Zhang et al., 2021). Additionally, there are other receptors which can directly bind to LC3 without ubiquitination, thus initiating mitophagy, which mainly includes the Nip3-like protein X (NIX)/BCL2-interacting protein 3-like (BNIP3L) receptor, BCL2-interacting protein 3 (BNIP3) receptor, FUN14 domain containing 1 (FUNDC1) receptor (Villa et al., 2018; Ma et al., 2020; Poole et al., 2021).

Under physiological conditions, autophagy is capable of removing the abnormally aggregated proteins and degenerated subcellular organelles, while excessive autophagy may result in massive and unnecessary cell death (Wang Y. et al., 2020). After CI/RI, fluorescence results show that PINK1 accumulates on OMM and Parkin translocation occurs in the penumbra of rat cortex, and the levels of other related autophagy proteins such as LC3 and Beclin1 are elevated (Lan et al., 2018). Researchers have found that promoting mitophagy via PINK1/Parkin could decrease the accumulation of damaged mitochondria and ameliorate neuronal injury during CI/RI (Wu X. et al., 2018; Ma et al., 2020; Wang H. et al., 2020; Mao et al., 2022). Wu X. et al. (2021) found that NIX degradation leads to mitophagy deficiency in ischemic brains, indicating that NIX may be a potential therapeutic target for ischemic stroke. Overexpression of FUNDC1 inhibits apoptosis and improves mitochondrial function against CI/RI (Cai et al., 2021). In myocardial ischemia/reperfusion, hypoxic preconditioning could induce FUNDC1-dependent mitophagy to resist ischemia/reperfusion injury (Zhang W. et al., 2017). This indicates that similar mechanisms may exist in CI/RI. The dynamic balance between these three processes of MQC is essential for maintaining mitochondrial homeostasis and function.

## 3. PANoptosis/ferroptosis in CI/RI

### 3.1. PANoptosis

**FIGURE 2:**
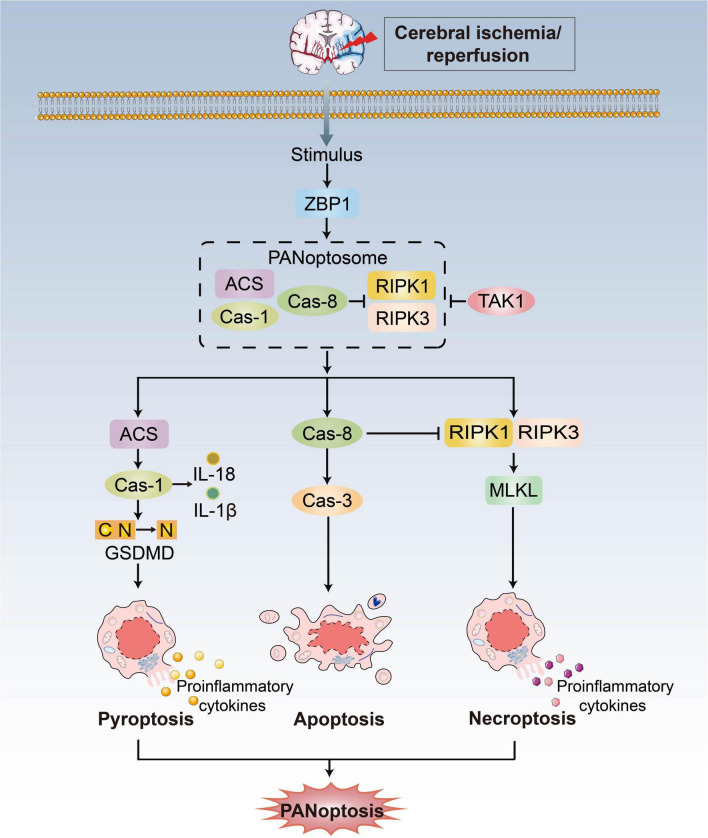
PANoptosis pathways. Exposure to stimulus during CI/RI can lead to the initiation of the apical sensors, such as ZBP1, which then induces the activation of proteins involved in pyroptosis, apoptosis, and necroptosis to form the ZBP1-PANoptosome and mediate PANoptosis. Three arms of cell death are executed by GSDMD family proteins (pyoptosis), caspase-3/7/8 (apoptosis) and MLKL (necroptosis). TAK1 could block formation of the PANoptosome and induction of PANoptosis.

Initially, pyroptosis, apoptosis and necroptosis were considered different and independent (Chen X. et al., 2022). The crosstalk between these pathways has therefore led to the establishment of the concept of PANoptosis, defined as an inflammatory PCD pathway with key features of pyroptosis, apoptosis, and necroptosis that cannot be accounted for by any of these three PCDs pathways alone (Figure 2; Kuriakose et al., 2016; Kesavardhana et al., 2017; Malireddi et al., 2018, 2019, 2020a). Pathogen- or pharmacologically mediated obstruction of survival signaling acts as a key danger signal to trigger the assembly of PANoptotic cell death complexes (Wang and Kanneganti, 2021). Recent progress has shown that receptor-interacting protein kinase (RIPK)1/RIPK3, Fas-associated protein with a death domain (FADD), caspase-8 and apoptosis-associated speck-like protein containing a caspase recruitment domain (ASC) are the master regulators to form a PANoptosome and then activate PANoptosis (Malireddi et al., 2019), which is equivalent to providing a molecular scaffold that allows engagement of key pyroptotic, apoptotic, and necroptotic machinery (Briard et al., 2021; Chen X. et al., 2022).

Z-DNA-binding protein (ZBP1) acts as an innate immune sensor to activate all three pathways and inflammation that contemporaneously engages key molecules from pyroptosis, apoptosis and necroptosis. Upon sensing stimulus, ZBP1 activation leads to its interaction with RIPK1/RIPK3, FADD and caspase-8 to form cell death signaling scaffolds (Malireddi et al., 2019). Additionally, growth factor beta-activated kinase 1 (TAK1) acts as a master switch for PANoptosis quiescence (Malireddi et al., 2019). TAK1 inhibition/deletion leads to the activation of apoptosis, pyroptosis, and necroptosis (Malireddi et al., 2018, 2020b; Orning et al., 2018; Sarhan et al., 2018). In the absence of external stimuli, TAK1 deficiency causes loss of cellular homeostasis and unleashes inflammatory signaling and PANoptosis (Malireddi et al., 2020b). There are still unanswered questions concerning the mechanistic details of PANoptosis, even though ZBP1 and TAK1 are known as regulators.

### 3.2. Ferroptosis

Ferroptosis is a distinct PCD type characterized by lipid peroxidation relying on ROS generation and severe iron overload (Yang and Stockwell, 2008; Dixon et al., 2012). This pathway is essential in neuronal cell death (Lu et al., 2017; Wu J. R. et al., 2018). Morphologically, ferroptosis causes reduction or vanishing of mitochondria crista, condensed mitochondrial membrane densities, and OMM rupture (Xie et al., 2016; Wang H. et al., 2020), a unique feature that is distinguishable from other forms of cell death (Dixon et al., 2012). Emerging evidence suggests that stroke is associated with iron buildup, lipid peroxidation, and a reduction of glutathione (GSH) and glutathione peroxidase 4. (GPX4). In neurons, GPX4 can inhibit excessive lipid peroxidation. Hence GPX4 activity inhibition triggers ferroptosis (Gaschler et al., 2018; Ingold et al., 2018; Kang et al., 2018). The lethal metabolic imbalance resulting from GSH depletion or inactivation of GPX4 is the executor of ferroptosis within the neural cell (Stockwell et al., 2017). The injury of the cystine/glutamate antiporter system (system Xc-), which consists of solute carrier family 3 member 2 (SLC3A2) and solute carrier family 7 member 11 (SLC7A11), lessens GSH production and GPX4 activation, resulting in lipid peroxidation of polyunsaturated fatty acids (PUFAs) and the accumulation of PUFAs-O-OH that can form lipid ROS. In contrast, Fe^2+^ ions are present in large quantities, which bind to PUFAs-O-OH and then initiate lipid ROS by the Fenton reaction, leading to iron death and neuronal damage (Cao and Dixon, 2016; Yang et al., 2016; Yang and Stockwell, 2016). PANoptosis and ferroptosis differ in morphological characteristics, signaling pathways, inhibitors/key regulators, and mitochondrial association (Table 1).

**TABLE 1:** Hallmarks of four types of programmed cell death (PCD).

	Pyroptosis	Apoptosis	Necroptosis	Ferroptosis
Morphological changes	Cellular swelling, membrane rupture, and cellular contents flowing out	Wrinkled cells, nuclear condensation, cell membrane ectropion, and apoptotic body formation	Cell enlargement, cellular swelling, and membrane rupture	Shrunken mitochondria, mitochondrial membrane condensation, mitochondria crista reduction, and outer mitochondrial membrane rupture, and mitochondria fragment
Signaling pathway	Pyroptosis pathway: caspase1-dependent pyroptosis and caspase1-independent pyroptosis	Apoptosis pathway: extrinsic pathway (receptor-mediated) and intrinsic pathway (mitochondria-mediated); p53-mediated apoptosis pathway	Necroptosis pathway and TNF pathway	Ferroptosis pathway and p53 pathway
Inhibitors	VX765 (Chen Y. et al., 2022)	Z-VAD FMK (Yu et al., 2022)	Necrostatin-1 (Dong et al., 2022)	Ferrostatin-1 (Liu et al., 2020c), liproxstatin-1 (Fan et al., 2021), DFO (Shen et al., 2022)
Key regulators	GSDMD, caspase-1/4/5/11, IL-1β, and IL-18	Fas/TNFR/TRAILR, caspase-3/8/9, Bax/Bcl-2, Cyt C, and APAF-1	RIP1, RIP3, MLKL, and Fas/TNFR	GPX4, JAK, SLC7A11, ACSL4, FPN, p53, and NADPH oxidase
Mitochondrial dysfunction	ROS and Ca^2+^ overload	Cyt C releases, BAX/BAK, and Bcl family protein interact	ROS burst, Drp1 activation, and mitochondrial fisson	Mitochondrial lipid peroxidation and Ca^2+^ overload

### 3.3. PANoptosis in CI/RI

The recent progress in understanding of the extensive crosstalk between different PCDs and signaling cascades unequivocally establishes the existence of multifaceted signaling platforms. It is well established that pyroptosis, apoptosis, and necroptosis occur simultaneously during CI/RI in diverse passage cell lines or primary neurons. Moreover, PANoptosis can contribute to neuroinflammation, which has widespread repercussions on the body. Since the components of the PANoptosome are widely implicated in neurological disorders, an improved understanding of the molecular underpinnings of the PANoptosis will be able to inform the development of new and improved therapeutic strategies (Malireddi et al., 2020b).

There are many central nervous systems (CNS) diseases characterized PANoptosis (Yuan and Yankner, 2000; McKenzie et al., 2020; Yan et al., 2021), which is generally associated with inflammatory reactions (Pender and Rist, 2001; Degterev et al., 2019; Lünemann et al., 2021). The inflammasome (Friedlander et al., 1997), caspase-8 (Krajewska et al., 2011), RIPK1 (Xu et al., 2018; Degterev et al., 2019) and other core components of the PANoptosome, are implicated in neuronal death (Fricker et al., 2018). Inflammation and immune system activation are often involved in the CI/RI pathophysiology, which can cause serious brain damage (Chamorro et al., 2016; Lambertsen et al., 2019; Shi et al., 2019; Zhang F. et al., 2022). In the existing studies of PANoptosis, the expression of cell death and the pathophysiological mechanism related to inflammation in IS are similar to the phenotype and mechanism, which provides basic evidence for the possible existence of PANoptosis and PANoptosomes (Yan et al., 2022). Otherwise, glial cells have been reported to interfere with these three forms of cell death after being stimulated by injury (Zhao et al., 2017; Xu et al., 2019; Naito et al., 2020; Liu X. et al., 2022), which overlaps with the inflammation-related and immune-related reports of existing studies of PANoptosis (Yan et al., 2022). Moreover, studies have shown that some molecules can simultaneously interfere with two PANoptosis components under CI/RI. RIPK3, as the key molecule of necroptosis, can interact with the Jun N-terminal kinase-mediated inflammatory signaling pathway, which is closely related to neuronal apoptosis (Hu et al., 2020). Blocking of thromboxane A synthase/thromboxane A2/thromboxane prostanoid signal can inhibit apoptosis and pyroptosis concurrently (Chueh et al., 2020). Moreover, the nucleotide oligomerization domain-like receptors with caspase activation and recruitment domain 4 (NLRC4) inflammasome complex can simultaneously regulate apoptosis and pyroptosis (Poh et al., 2019). Hence, PANoptosis induced by CI/RI could be regulated and intervened simultaneously.

Although there is no study on the PANoptosome in CI/RI, the existing data of the components that make up a PANoptosome are highly expressed in the brain. Studies have shown that inhibiting TAK1 can reduce neuronal death induced by CI/RI (Neubert et al., 2011; Wang L. et al., 2019; Wu et al., 2020). Additionally, TAK1 affects the microglia’s function and interacts with an inflammatory pathway to activate neuronal apoptosis and pyroptosis (Gong et al., 2015; Zeyen et al., 2020). Furthermore, it is vital in the interaction between necroptosis and apoptosis of neurons during CI/RI (Naito et al., 2020). All these findings show that molecules like TAK1 may regulate PANoptosomes in CI/RI.

### 3.4. Ferroptosis in CI/RI

There is considerable evidence that ferroptosis plays a significant role in CI/RI pathogenesis. Research indicates that ferroptosis occurs mainly in neurons and exacerbates CI/RI (Guan et al., 2019; Yuan et al., 2021; Liu W. et al., 2022). The CI/RI pathogenesis results in increased vulnerability to oxidative stress and ATP production, which is impeded to maintaining metabolic activity and the activity of system Xc-. Meanwhile, neuronal membranes are rich in PUFAs, which are easy to lipid hydroperoxides and induce ferroptosis (Conrad and Pratt, 2019).

Moreover, under CI/RI conditions, iron accumulation in affected brain areas is the key mediator of neuronal damage and death (Castellanos et al., 2002). Iron chelation therapy, such as deferoxamine, has been shown to attenuate the cellular damage observed in the brains of experimental I/RI animal models (Prass et al., 2002; Hanson et al., 2009). Another study demonstrates that ferroptosis inhibition by GPX4 provides protective mechanisms against neurodegeneration (Zou and Schreiber, 2020; Li et al., 2022). Alim et al. (2019) reported that pharmacological selenium could augment GPX4 expression, which inhibits ferroptosis and protect neurons from CI/RI in C57BL/6 mice. Furthermore, dihydromyricetin (DHM) represses ferroptosis by SPHK1/mTOR signaling pathway inhibition, thereby alleviating CI/RI, suggesting that DHM may be a candidate drug for CI/RI treatment (Xie J. et al., 2022). Additionally, CI/RI-related neuronal damage can be rescued by ferroptosis inhibitors such as liprostatin-1 and ferrostatin-1, strongly suggesting a direct involvement of ferroptosis in CI/RI (Tuo et al., 2017). Primarily, additional research into the involvement of ferroptosis in CI/RI is required. Ferroptosis is the primary cause and a potential treatment for IS and other cerebrovascular disorders.

## 4. Mitochondrial dysfunctions in cell death

### 4.1. Mitochondrial dysfunctions and pyroptosis

Mitochondrial ROS (mtROS) has long been considered a key signaling molecule for pyroptosis since it promotes the efficiency of the GSDMD (Gasdermin D) cleavage by caspase-1 (Wang C. et al., 2019). Active GSDMD forms pore permeabilizes, leading to pyroptosis. In turn, active GSDMD and inflammasome can cause MOMP (mitochondrial outer membrane permeabilization), which induces mitochondrial dysfunctions and forms extensive crosstalk between pyroptosis and mitochondrial apoptosis (Rogers et al., 2019; Tsuchiya et al., 2019). Additionally, Yeon et al. (2017) showed that phospholipid oxidation and accumulation of oxidized phosphatidylcholine during cell injury could induce the production of mtROS, which then activates the NLRP3 inflammasome. The mtROS, mitochondrial Ca^2+^ and mitochondrial destabilization can induce NLRP3 inflammasome activation and activate pyroptosis (Yeon et al., 2017; Yu et al., 2019). Suppression of mitochondrial mitophagy also slows the pyroptosis progression (Yu et al., 2019).

### 4.2. Mitochondrial dysfunctions and apoptosis

Mitochondria are key factors in triggering apoptosis. The intrinsic pathway is related to mitochondria (Wei et al., 2001). Upon induction of mitochondrial apoptosis effectors, MOMP is driven by pro–apoptotic members of the BCL–2 family of proteins (prominently BAX and BAK). Activation of the pro–apoptotic effectors BAX and BAK are usually essential for MOMP and cell death (Wei et al., 2001). Under normal conditions, inactive BAX localizes to the cytoplasm and inactive BAK to the mitochondria. Once activated, they can directly bond to a subclass of BH3–only proteins like tBID (truncated active BID) (Letai et al., 2002), and BAX will accumulate in the mitochondria (Edlich et al., 2011; Schellenberg et al., 2013; Todt et al., 2015). BAX/BAK commits the release of soluble proteins-Cyt C, which activates the downstream caspase cascade (Wei et al., 2001).

Most Cyt C resides within mitochondrial cristae and is regulated by cristae junctions (van der Laan et al., 2016). Mitochondria, the dynamic organelles, can constantly undergo fission cycles and fusion by mitochondrial fission protein Drp1 (Frank et al., 2001; Bhola et al., 2009) to remodel mitochondrial cristae, which has been proposed to facilitate Cyt C release. Mdivi-1 is a Drp1 inhibitor that prevents mitochondria division and Bax-mediated MOMP during apoptosis (Tanaka and Youle, 2008; Nhu et al., 2021). Cyt C also induces tumor gene p53. Recent investigations show that the p53 protein can defect MOMP by forming an inhibitory complex with the Bcl-2 family protein, leading to Cyt C release (Bakthavachalam and Shanmugam, 2017). Meanwhile, MOMP causes the release of proteins, including the second mitochondria-derived activator of caspase (SMAC) and OMI/HTRA2 that block the caspase-7/9 inhibitor X-linked inhibitor of apoptosis protein (XIAP), facilitating apoptosis (Bock and Tait, 2020). Even in the absence of caspase activity, cells usually die following BAX/BAK-dependent MOMP, which releases mtDNA by mitochondrial inner membrane permeabilization (MIMP) and then activates cGAS-STING signaling during apoptosis (Riley et al., 2018). Elevated levels of mtROS also induces cell oxidative stress and destroy the cellular structure and MOMP. ROS are involved in both caspase-dependent and caspase-independent pathways, which is an important bridge between these two apoptosis types (Chen C. et al., 2020). The extrinsic pathway is activated at the plasma membrane by death receptor ligands binding to their related receptors, interacting with the pro-caspase-8 and forming a death-induced signaling complex (DISC), leading to activation of caspase-8 and activate pro-caspase-3/7. Caspase-8 is the crosstalk to the mitochondrial pathway (Datta et al., 2020).

### 4.3. Mitochondrial dysfunctions and necroptosis

Necroptosis is morphologically characterized by electron-lucent cytoplasm, cell swelling, shrinking of organelles, cell membrane rupture, dilation of the perinuclear space and spilling of intracellular damage-associated molecular patterns (DAMPs) out of the cell (Kaczmarek et al., 2013), which can trigger an inflammatory response (Chen et al., 2019; Miyake et al., 2020). RIPK1-RIPK3-MLKL necrosome is essential in necroptosis through mitochondria (Zhe-Wei et al., 2018; Zhou et al., 2018).

TNF-α (tissue necrosis factor-alpha) binds to TNFR on cell surface and transmits death signals via RIPK1 and RIPK3, forming RIPK1-RIPK3-MLKL necrosome. RIPK3 and MLKL phosphorylation upregulate phosphoglycerate mutase family member 5 (PGAM5) expression on the mitochondrial membrane. PGAM5 can increase CypD phosphorylation, which obligated endothelial cells to undergo necroptosis by augmenting mPTP opening. Blocking the RIPK3-PGAM5-CypD signal pathways can suppress mPTP opening and interrupt necroptosis (Zhou et al., 2018). PGAM5 enters the cytoplasm to collaborate with Drp1 (Feng et al., 2020), which inhibits glutathione production and disrupts mitochondrial metabolism, leading to reduced free radical removal capacity and increased mtROS (Wang et al., 2012; Zhou et al., 2018; Xiao et al., 2020). Moreover, the necrosome can also affect metabolic enzymes glutamate dehydrogenase 1 (GLUD1), glycogen phosphorylase (PYGL), and pyruvate dehydrogenase (PDH) to promote the production of mtROS (Zhang et al., 2009; Han et al., 2018; Yang et al., 2018; Zhao et al., 2021). In turn, the released mtROS can facilitate the RIPK1 autophosphorylation and RIPK3 recruitment, which are critical for necroptosis (Schenk and Fulda, 2015; Zhang Y. et al., 2017).

### 4.4. Mitochondrial dysfunctions and ferroptosis

Ferroptosis is characterized morphologically by abnormal mitochondrial architecture, including mitochondrial fragmentation, shrunken mitochondria, rupture of OMM and vanished mitochondrial cristae (Xie et al., 2016; Miyake et al., 2020; Wang H. et al., 2020). Abnormal mitochondrial architecture, including mitochondrial fragmentation, shrunken mitochondria and rupture of the mitochondrial outer membrane, and vanished mitochondrial cristae, is regarded as the typical morphological characteristic of ferroptosis.

Currently, whether mitochondria have an impact on ferroptosis remains a controversial research topic. Furthermore, recent nervous system studies have revealed that the burst of lethal mtROS and the accumulation of lipid peroxidation products affect proteins related to iron metabolism in the mitochondrial membrane, which is the main reason to mediate ferroptosis in neurons (Gao and Chang, 2014; Xie et al., 2016; Liu et al., 2020a,b). Meanwhile, iron overload, one of the mechanisms of ferroptosis, has been shown to trigger mPTP opening and necroptosis by ROS accumulation (Tian et al., 2020).

Mitochondria are vital in ferroptosis induced by the lack of cysteine. Cysteine deprivation induces the decomposition of glutamine (Gln), a non-essential amino acid that serves as the major respiratory fuel for energy production and lipid biosynthesis. Gln drives the hyperpolarization of MMP and feeds the tricarboxylic acid (TCA) cycle (Gao et al., 2019), thereby increasing mitochondrial respiration by ETC in consequence, augmenting levels of mitochondrial ROS to initiate the Fenton reaction (Gao et al., 2019; Bock and Tait, 2020). Additionally, increased mtROS induced by Gln facilitates the overload of mitochondrial Ca^2+^ (Maher et al., 2018) and the mPTP opening, causing dissipation of the mitochondrial transmembrane potential and subsequent ATP depletion (Bernardi and Di Lisa, 2015; Ying and Padanilam, 2016; Novgorodov et al., 2018). Furthermore, the mitochondrial VDACs were proved to be a potential target of erastin by decreasing ΔΨm (Yagoda et al., 2007). Opening VDACs leads to an increase in MMP, and then mtROS generates (Yagoda et al., 2007; DeHart et al., 2018). Mitochondrial ferritin (FtMt), an iron-storage protein, has been reported to protect mitochondria from iron-induced oxidative damage, presumably through the chelation of potentially harmful excess free iron (Nie et al., 2005; Gao and Chang, 2014). It also participates in the regulation of iron distribution between cytosol and mitochondrial contents. FtMt has been shown to significantly inhibit the cellular labile iron pool (LIP) level, ROS and subsequent ferroptosis by the Fenton reaction (Yarmohammadi et al., 2021; Boag et al., 2022).

**FIGURE 3:**
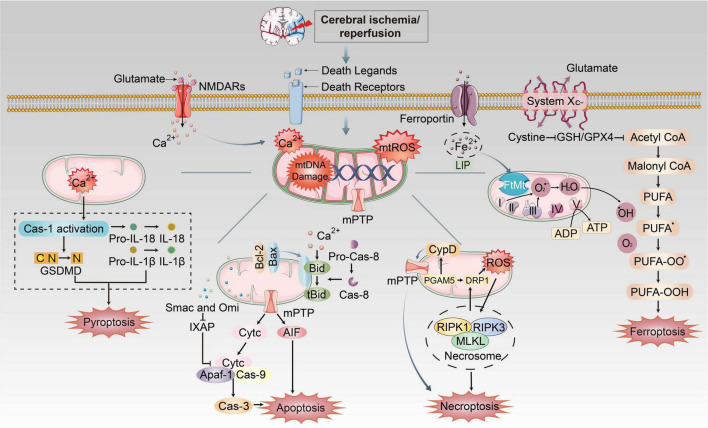
The mechanism of mitochondrial dysfunctions induced PANoptosis and ferroptosis during CI/RI. Mitochondria play essential roles in pathological conditions after ischemic stroke and reperfusion. During ischemia, oxygen–glucose deprivation will cause ATP consumption and the bind of death ligands to death receptors on the membrane. Na^+^/K^+^ ATPase pump failure that induces depolarization of neuronal membranes and extreme release of glutamate, burst of ROS, free radical damage, Ca^2+^ homeostasis disorder and EAA toxicity, etc. Iron was released into the brain parenchyma, which accelerates lipid ROS accumulation and ferroptosis via Fenton reaction. The extracellular death ligands bind to death receptors, which triggers the recruitment of FADD or TRADD to induce apoptosis and necroptosis, respectively. These mechanisms cause mitochondrial dysfunctions such as formation of mPTP, burst of mtROS, mitochondrial Ca^2+^ overload and mtDNA damage, which could execute different cell death pathways.

During CI/RI, mitochondrial dysfunctions play essential roles in pathological conditions in PANoptosis and ferroptosis, as previously stated (Figure 3). The mtROS burst, mtDNA defects, mPTP formation, mitochondrial Ca^2+^ overload and iron dyshomeostasis are central parts of cell death. It has been demonstrated that mitochondrial dysfunctions are closely associated with various PCDs in the pathophysiological process of CI/RI, and therefore the rational use of these mechanisms in the biomedical field to address mitochondria as the target for drug development and therapeutic strategies to ameliorate PCD in IS could be a promising option.

## 5. Therapeutical potential of targeting the mitochondrial dysfunctions against CI/RI in ischemic encephalopathy

Mitochondrial dysfunctions are the main feature seen during the initiation of stroke pathophysiology. Consequently, targeting mitochondria dysfunctions represents a promising strategy to attenuate CI/RI-induced diseases (Carinci et al., 2021). Interventions that directly target mitochondrial dysfunctions by alleviating different cell death during CI/RI are summarized in the present review (Table 2).

**TABLE 2:** Therapeutic strategy by targeting mitochondrial dysfunctions to inhibit cerebral ischemia/reperfusion injury (CI/RI).

Cell deaths	Interventions	Model	Subjects	Mitochondrial associated targets	Functions	References
Pyroptosis	Idebenone	OGD/R and tMCAO	PC12 cells, BV2 cells, and rat	mtDNA and mtROS	Idebenone suppresses activation of NLRP3 and ameliorates NLRP3-mediating damage in I/R.	Peng et al., 2020
	Medioresinol	OGD, tMCAO	bEnd.3 cells, BMVECs, and mice	mtROS	MDN decreases mtROS through PPARα/GOT1 axis and ameliorate the pyroptosis and ischemic brain injury.	Wang Y. et al., 2021
	Umbelliferone	MCAO/R	Rat	ROS/TXNIP	UMB protects focal cerebral ischemic through the inhibition of TXNIP/NLRP3 inflammasome and activation of PPAR-γ.	Wang et al., 2015
Apoptosis	miR-668 inhibitor	tMCAO/R	Rat	Drp1, mtROS, Bax/Bcl-2	The miR-668 inhibitor prevents neuronal apoptosis in CI/RI by modulating mitochondrial function and regulating NLRP3 signaling.	He and Zhang, 2020
	Candesartan	OGD/R	PC12 cells	Bax	Candesartan inhibits apoptosis by downregulation of Bax and cleaved caspase-3 in OGD/R-PC12 cells.	Ding et al., 2022
	Edaravone dexborneol	Four-vessel occlusion (4-VO)	Rat	Bax/Bcl-2	Edaravone-Dexborneol alleviates cerebral ischemic injury via reduction of apoptosis and neuron damage.	Zhang W. et al., 2022
	Tong-Qiao-Huo-Xue-Decoction formula	MCAO	Rat	Bax/Bcl-2	TQHXD protects neurons from I/R damage and prevents apoptosis.	Yuan et al., 2022
	CsA	BBCAO/R	Rat	Bax/Bcl-2	CsA decreases Bax/Bcl-2 ratio as well as caspase-3 activation.	Fakharnia et al., 2017
Necroptosis	CsA	BBCAO/R	Rat	mPTP	CsA inhibits mPTP opening and reduces RIP1 and RIP3 levels.	Fakharnia et al., 2017
	Infliximab	tMCAO	Rat	Mitochondrial membrane potential	Infliximab ameliorates endothelial necroptosis and reduces mitochondrial damage, cytoplasm transparency, and BBB permeability.	Chen et al., 2019
	rhTrx-1	MCAO	C57BL/6 mice	Mitochondrial membrane potential	rhTrx-1 provides neuroprotection in IS-induced microglial neuroinflammation by inhibiting RIPK1 expression	Jiao et al., 2020
Ferroptosis	UBIAD1	MCAO/R, OGD/R	Rat, primary neurons	Mitochondrial protein complexes	UBIAD1 modulates I/R-mediated ferroptosis by restoring mitochondrial dysfunctions and enhances antioxidative capacities.	Huang et al., 2022
	FtMt	MCAO/R	Mice	FtMt	FtMt protects against CI/RI-induced ferroptosis.	Wang P. et al., 2021
	Ferrostatin-1	t-BHP treatment	PC12 cells	Mitochondrial membrane potential, ATP production, and mtROS	Ferrostatin-1 reverses ferroptosis-induced mitochondrial dysfunctions	Wu C. et al., 2018

Idebenone is a well-appreciated mitochondrial protectant in cerebral ischemia and reperfusion. Peng et al. (2020) found that mitochondrial dysfunctions in OGD/R leads to accumulation of oxidized mtDNA and mtROS generation, dramatically augments inflammation in BV2 and PC12 cells. Idebenone inhibits the process and attenuates cerebral inflammatory injury in ischemia and reperfusion by dampening NLRP3 inflammasome activity. MicroRNAs (miRNAs) are a group of small non-coding RNA molecules that regulate gene expression at the post-transcriptional level. The miR-668 expression level has been reported to be altered under ischemic conditions in cell culture and animal models (Chun et al., 2018). The miR-668 inhibition prevents neuronal apoptosis in CI/RI by modulating mitochondrial functions such as reduction of Drp1 and melioration of the expression of Bax/Bcl-2 protein (He and Zhang, 2020). CypD is a prominent mediator of mPTP, which leads to mitochondrial swelling and dissipation of MMP on necroptosis, autophagy, and apoptosis beyond CI/RI. Cyclosporine-A (CsA) is a potent inhibitor of CypD. Fakharnia et al. (2017) found that CsA reduces necroptosis markers, RIP1 and RIP3. Furthermore, the Bax/Bcl-2 ratio and caspase-3 activation, as the executioner of apoptosis, noticeably decreases by CsA pretreatment (Fakharnia et al., 2017). It suggests that CsA-mediated CypD inhibition may provide a promising therapeutic potential for protecting against CI/RI-mediated mitochondrial dysfunctions. UBIAD1 is a newly identified antioxidant enzyme that acts on the Golgi apparatus membrane and mitochondria (Nakagawa et al., 2010; Mugoni et al., 2013). Upregulated UBIAD1 protects against brain tissue damage and neuronal death by rescuing the morphology and bio functions of the mitochondria and Golgi apparatus in CI/RI, thus alleviating I/R-mediated lipid peroxidation and ferroptosis (Huang et al., 2022). Moreover, the rescue of impaired mitochondrial as a possible mechanism of regulating ferroptosis neuronal death is a potential treatment strategy for IS. FtMt is a key mitochondrial iron storage protein that protects cells from iron-dependent oxidative damage rather than being directly related to cellular iron levels (Nie et al., 2005). Mice lacking FtMt experience more severe brain damage and neurological deficits, accompanied by typical molecular features of ferroptosis after CI/RI. Conversely, FtMt overexpression reverses these changes, which limits CI/RI-induced iron overload and iron-dependent lipid peroxidation and suppresses ferroptosis in the penumbra (Wang P. et al., 2021). FtMt may be a potential therapeutic target in ischemic stroke.

In conclusion, we have discussed the therapeutic potential of targeting mitochondrial dysfunctions on PCDs in IS or CI/RI and the associated mechanisms. New therapeutic strategies that target mitochondrial dysfunctions may be used to mitigate the devastating effects of CI/RI.

## 6. Conclusion and perspective

As stated above, mitochondrial dysfunctions highlight the essential role of cell death during CI/RI, providing us with a more comprehensive and profound understanding of pathogenesis of which is associated with mitochondrial oxidative stress, Ca^2+^ overload, iron dyshomeostasis, mtDNA defects and MQC disruption, eventually triggering programmed cell deaths (Giorgi et al., 2018). Emerging researches have indicated that mitochondrial molecules such as mPTP, FtMt, proteins of MQC, Bax/Bcl-2 might be the crosstalk between mitochondrial dysfunctions and PCD pathways. These markers have diagnostic or prognostic values for patients with IS.

Furthermore, the study of mitochondrial dysfunctions is conducive to developing potential molecular therapeutic strategies that target CI/RI. The natural inhibitors or small molecules modifying mitochondrial dysfunctions are of high efficacy for the treatment and prevention of the cell death pathways (Egawa et al., 2017). For example, CsA, a potent inhibitor of CypD and mPTP, could decrease Bax/Bcl-2 ratio as well as caspase-3 activation for apoptosis intervention, while it also reduces RIP1 and RIP3 levels to suppress necroptosis (Fakharnia et al., 2017). Currently, stem cells have shown the ability to transfer mitochondria to the injured cells, which helps to protect mitochondria and revive cell energetics (Sarmah et al., 2018). Additionally, traditional Chinese medicine (TCM) acts as a promising candidate in breaking the vicious cycle between mitochondrial dysfunctions and PCD pathways, improving the quality of life of the stroke patients. It provides a multiple-target approach rather than a single-target approach and thus can target multiple pathways involved in CI/RI at once. Taken together, further studies targeting mitochondrial dysfunctions will provide novel opportunities for the treatment of IS.

However, some limitations exist in the current studies. Firstly, the molecular mechanisms underlying mitochondria-targeted cell death pathways has not been fully elucidated. Secondly, under different pathological injury states of IS, the role of mitochondria is dissimilar. Additionally, the activation of molecular executioner signatures of pyroptosis, apoptosis, necroptosis, and ferroptosis are not required simultaneously in an individual cell for a cell death process to fit (Gullett et al., 2022), which remains optimal time window of intervention unclear. Thirdly, current studies might be limited by the lack of clinical tests to assess the status of mitochondrial dysfunctions. Besides, other clinical biomarkers have poor sensitivity and specificity to predict the outcome of CI/RI.

Consequently, further studies are recommended to develop novel and targeted mechanisms centered on the mitochondrial dysfunctions to improve prognosis in patients with CI/RI. Genome, transcriptome, proteome, epigenome sequencing techniques and radiomics can identify the molecular heterogeneity that reveals the crosstalk between mitochondrial dysfunctions and PCDs in a patient-specific manner. Meanwhile, investigating the expression of PCDs markers as well as mitochondrial morphological changes and dysfunctions at different phases of functional recovery after CI/RI can provide valuable insights into best time-window of treatment for CI/RI. Furthermore, we anticipate that in clinical trials, combining ultrasound, CT, serum markers and other technologies can effectively improve the diagnostic accuracy of mitochondrial dysfunctions in the early stage, and guide the clinical treatment. We hold the view that the in-depth study of mitochondrial dysfunctions-induced PANoptosis and ferroptosis would provide new perspectives, potential therapeutic targets for ischemic stroke and other ischemia-induced diseases of CNS.

## Author contributions

ZM and JG conceived and supervised the work and revised the manuscript. RS drafted the initial manuscript. DL, JL, and GW provided some positive suggestions and amended the manuscript. All authors contributed to manuscript revision, read, and approved the submitted version.
